# PDE based scheme for multi-modal medical image watermarking

**DOI:** 10.1186/s12938-015-0101-x

**Published:** 2015-11-25

**Authors:** N. Aherrahrou, H. Tairi

**Affiliations:** LIIAN, Department of Informatics, University Sidi Mohamed Ben Abdellah, Fez, Morocco

**Keywords:** Watermarking, PDE, ROI, RONI, Authenticity, Integrity

## Abstract

**Background:**

This work deals with copyright protection of digital images, an issue that needs protection of intellectual property rights. It is an important issue with a large number of medical images interchanged on the Internet every day. So, it is a challenging task to ensure the integrity of received images as well as authenticity. Digital watermarking techniques have been proposed as valid solution for this problem.

**Methods:**

It is worth mentioning that the Region Of Interest (ROI)/Region Of Non Interest (RONI) selection can be seen as a significant limitation from which suffers most of ROI/RONI based watermarking schemes and that in turn affects and limit their applicability in an effective way. Generally, the ROI/RONI is defined by a radiologist or a computer-aided selection tool. And thus, this will not be efficient for an institute or health care system, where one has to process a large number of images. Therefore, developing an automatic ROI/RONI selection is a challenge task. The major aim of this work is to develop an automatic selection algorithm of embedding region based on the so called Partial Differential Equation (PDE) method. Thus avoiding ROI/RONI selection problems including: (1) computational overhead, (2) time consuming, and (3) modality dependent selection.

**Results:**

The algorithm is evaluated in terms of imperceptibility, robustness, tamper localization and recovery using MRI, Ultrasound, CT and X-ray grey scale medical images. From experimental results that we have conducted on a database of 100 medical images of four modalities, it can be inferred that our method can achieve high imperceptibility, while showing good robustness against attacks. Furthermore, the experiment results confirm the effectiveness of the proposed algorithm in detecting and recovering the various types of tampering. The highest PSNR value reached over the 100 images is 94,746 dB, while the lowest PSNR value is 60,1272 dB, which demonstrates the higher imperceptibility nature of the proposed method. Moreover, the Normalized Correlation (NC) between the original watermark and the corresponding extracted watermark for 100 images is computed. We get a NC value greater than or equal to 0.998. This indicates that the extracted watermark is very similar to the original watermark for all modalities.

**Conclusion:**

The key features of our proposed method are to (1) increase the robustness of the watermark against attacks; (2) provide more transparency to the embedded watermark. (3) provide more authenticity and integrity protection of the content of medical images. (4) provide minimum ROI/RONI selection complexity.

## Background

The fast development of use of Internet and wireless networks provide easy access, handle and exchange of medical images. They also allow easy manipulation and replication. It is fairly easy to intercept or tamper sensitive medical data when the public network is being used for applications as in the case of teleradiology (telesurgery or telediagnosis). Thus there is urgent need of security measures in medical information system. Digital watermarking techniques have been proposed as valid solution for this problem [1–5]. The idea of watermarking is to embed secret information (watermark) inside an image, audio or video file to increase the digital data security.

Many watermarking techniques were proposed during the last few years to fulfill this requirement. These techniques are based on typical requirements of any digital watermarking scheme including imperceptibility and robustness criteria. Imperceptibility is defined as a watermark that should not introduce any perceptible artifacts into the original image. For the robustness it means that a watermark should not be removed after attacks.

Moreover these techniques require in case of medical images watermarking more authenticity and integrity. While authenticity ensures that the image belongs to the claimed patient and comes from the correct source. Integrity verifies that the image has not been modified [3–5]. To provide these security requirements, many watermarking techniques were proposed in the literature. These techniques can be classified into three categories of methods: Irreversible, reversible and region based watermarking methods. The irreversible methods include methods based on using classical watermarking techniques minimizing the distortion [6, 7]. This kind of methods is not acceptable in the medical field since the distortion applied to the original image by the watermarking process is not reversible. The reversible methods ensure that the embedded watermarks are removed once the watermarks have been detected and verified. Thus, the image can be retrieved in its original form [8–10]. Even if this kind of methods restores the watermarked images, they present some limitations. (1) It imposes the watermark removal before the diagnosis, and (2) it assumes a secured environment because, once the watermark is removed, the image is not protected anymore [11]. In addition, most reversible watermarking algorithms lack the tamper localization functionality, which is a desired property in the integrity verification of medical images [12].

Finally the region based watermarking methods separate medical images into two parts; A significant part, which is called Region Of Interest (ROI) and a part that does not contain any clinical findings, the region of non-interest (RONI) [12–14]. The selection of the ROI/RONI is varying. Some authors define RONI as the region of background corresponding to the black area inside an X-ray, a magnetic resonance imaging (MRI) image, or any other non-significant area of the image [15]. ROI can be defined as a rectangle, triangle, ellipse or polygon [10, 12–14]. For example, ROI is defined by using rectangles for MR-brain image [15], polygons for CT, MR, and US [12], logical ellipses for CT, US, X-ray, and MR images [16], and morphology operations [17].

Although the separation of ROI and RONI in medical imaging is not straightforward, it may require the approval of medical specialists including medical doctors and radiologists. Making such separation is sometimes very difficult and time-consuming, especially if one has to process a large number of images [1, 11]. To overcome these limitations, a few automatic selection algorithms have been proposed in literature [15–19]. The different proposed algorithms were previously found to be sensitive to noise and they are not equally suitable for different modality images [17]. Specific algorithms are only applied to specific modalities such as CT [16], MRI [15] or Ultrasound modalities [19].

Taken together, existing techniques are either manual or modality dependent and are thus inefficient and not widely useful. Therefore developing an automatic selection technique for multimodal medical images is a challenging task. In this paper, we aim to develop a scheme that can reasonably address all the above gaps and limitations. We propose here a new watermarking scheme, which satisfies the requirements of any medical images watermarking scheme including imperceptibility, robustness, authenticity and integrity. The proposed scheme is designed to be robust against various kinds of attacks by using a blind scheme in the Discrete Wavelet Transform (DWT)/Discrete Fourier Transform (DFT) transform domain. The authenticity is achieved by a robust watermark representing the logo of our University. The integrity of the medical images can be achieved by using a local watermark representing the textural part. The imperceptibility is achieved by embedding the watermarks in the texture and noise components while keeping the structural part intact.

In the following sections of this paper we included “related work” in section “Related work” as a survey of some recent related work. In “PDE, DWT and DFT models”, we introduce briefly the PDE, DWT and DFT decomposition for readers who are not familiar with these methods. “The proposed algorithm” presents more details about the proposed scheme. In “Evaluation and discussion” we evaluated the proposed approach. Finally in "Conclusion and discussions" we summarized and discussed our approach.

## Related work

Different types of watermarking methods have been previously reported for medical images to provide robust and effective digital watermarking scheme including imperceptibility, authenticity and integrity [12, 20, 21].

AlHaj and coworkers proposed a region based algorithm based on multiple watermarking in the frequency and spacial domains. Authenticity is provided by embedding a robust watermark, which represents the patient’s data in the RONI of the image using a blind scheme in the DWT-SWD transform domain. The integrity is provided by embedding local fragile watermark in the region of interest (ROI) of the image, using a reversible scheme in the spacial domain to identify and localize tampered areas. However, the quality of the watermarked images by Al Haj’s method needs to be improved [12].

The performance of watermarking in the spatial, Discrete Fourier Transform (DFT), Discrete Cosine Transform (DCT) and Discrete Wavelet Transform (DWT) coefficients is studied in [20]. The simulation results show that, among the frequency watermarking techniques, DFT was found to be very efficient.

In [21], a Robust watermarking method in DFT domain is proposed. The authenticity is achieved by embedding a generated watermark representing the electronic patient record (EPR) data into the magnitude of the middle frequencies of the discrete Fourier transform of the original medical image. The simulation results applied to medical imaging, using a set of 100 medical images in DICOM format and different types: CR, RF, MR and CT show that the proposed algorithm is robust against geometric distortions and common signal processing operations including Gaussian noise attack and JPEG compression. Moreover, the imperceptibility requirement for medical images is preserved achieving a PSNR greater than 49 dB. However, as drawbacks, the method proposed does not restore the electronic patient record (EPR) data to their text original format and the integrity of the watermarked image is not verified.

## PDE, DWT and DFT models

### PDE decomposition model

#### Presentation

Decomposing an image into meaningful components is an important and challenging inverse problem in image processing.

In the last few years, different algorithms have been proposed to decompose an image f into various components representing different information in the image.

Aujol and Chambolle [22], proposed a decomposition model that splits a grayscale image into three components: (a) the structure part, u ∈ BV^1^, which corresponds to the main large objects in the image; (b) a Texture part, v ∈ G^2^, which contains more details about the image and (c) the noise part, w ∈ E^3^, corresponding to isolated features (edges, ridges, corners) that do not belong to texture [22].

The decomposition model is performed by solving the following minimization:

1 $$\inf_{{({\text{u,v,w}}) \in {\text{X}}^{3} }} {\rm F}({\text{u}},{\text{v}},{\text{w}})$$

where,

2 $${\text{F}}({\text{u,v,w}}) = {\text{J}}({\text{u}}) + {\text{J}}*\left( {\frac{\text{v}}{\mu }} \right) + {\rm B}*\left( {\frac{\text{W}}{\delta }} \right) + \frac{1}{2\lambda }\left\| {{\text{f}} - {\text{u}} - {\text{v}} - {\text{w}}} \right\|_{X}^{2}$$

where J(u) is the total variation related to the extraction of the geometrical component, ${\text{J}}*\left( {\frac{\text{v}}{\mu }} \right)$, ${\text{B}}*\left( {\frac{\text{W}}{\delta }} \right)$ are the Legendre-Fenchel transforms^4^ of respectively J and B [23]. For the extraction of texture and noise components, The bound µ controls the G norm of the oscillating component v. The parameter λ controls the L^2^—norm of the residual f − u − v − w. The δ controls the E norm of the w component. X is the discrete Euclidean space ${\mathbb{R}}^{{^{{{\text{N}} \times {\text{N}}}} }}$ for images of size N × N.

To solve (1), Aujol and Chambolle consider the three following problems:v and w being fixed, they search for u as a solution of:

3 $$\inf_{{{\text{u}} \in {\text{X}}}} \left( {{\text{J}}({\text{u}}) + \frac{1}{2\lambda }\left\| {{\text{f}} - {\text{u}} - {\text{v}} - {\text{w}}} \right\|_{\text{X}}^{2} } \right)$$

u and w being fixed, they search for v as solution of:

4 $$\inf_{{{\text{v}} \in \mu {\text{B}}_{\text{G}} }} \left\| {{\text{f}} - {\text{u}} - {\text{v}} - {\text{w}}} \right\|_{\text{X}}^{2}$$

u and v being fixed, they search for w as solution of:

5 $$\inf_{{{\text{w}} \in \delta {\text{B}}_{\text{E}} }} \left\| {{\text{f}} - {\text{u}} - {\text{v}} - {\text{w}}} \right\|_{X}^{2}$$

For minimizing this function, Chambolle’s projection algorithms is used [23]. The Chambolle’s projection P on space $\uplambda {\text{B}}_{\text{G}}$^5^ of f is denoted ${\text{P}}_{{\lambda {\text{B}}_{\text{G}} }} ({\text{f}})$ and is solved by an iterative algorithm. This algorithm starts with P0 = 0 and for each pixel (i,j) and at each step n + 1 we have:

6 $${\text{P}}_{{{\text{i}},{\text{j}}}}^{{{\text{n}} + 1}} = \frac{{{\text{P}}_{{{\text{i}},{\text{j}}}}^{\text{n}} + \left( {\Delta {\text{div(P}}^{\text{n}} ) - \frac{\text{f}}{\lambda }} \right)_{{{\text{i}},{\text{j}}}} }}{{1 + \tau \left| {\Delta {\text{div(P}}^{\text{n}} ) - \frac{\text{f}}{\lambda }} \right|_{{{\text{i}},{\text{j}}}} }}$$

In [23] a sufficient condition ensuring the convergence of this algorithm is given: $\tau \le \frac{1}{8}$. The solution of (3) is simply given by:

7 $$\hat{u} = f - v - w - P_{{\lambda B_{G} }} \left( {f - v - w} \right)$$

The solution of (4) is simply given by:

8 $${\hat{\text{v}}} = {\text{P}}_{{{\rm \mu} {\text{B}}_{\text{G}} }} \left( {{\text{f}} - {\text{u}} - {\text{w}}} \right)$$

where P is the Chambolle projection on Space $\mu {\text{B}}_{\text{G}}$^6^ of v denoted by ${\text{P}}_{{\mu {\text{B}}_{\text{G}} }}$.

And the solution of (5) is given by:

9 $$\hat{w} = P_{{\delta B_{E} }} \left( {f - u - v} \right)$$

where P is the Chambolle projection on Space $\delta {\text{B}}_{\text{E}}$,^7^ of w denoted by ${\text{P}}_{{\delta {\text{B}}_{\text{E}} }}$.

The authors in [22] prove that the solution of minimizing (1) can be found by an iterative algorithm:

### Algorithm

1. 10 $${\rm Initialization} \; {\text{u}}_{0} = {\text{v}}_{0} = {\text{w}}_{0} = 0$$
2. 11 $${\text{Compute }} {\text{w}}_{{{\text{n}} + 1}} = {\text{P}}_{{\delta {\text{B}}_{\text{E}} }} \left( {{\text{f}} - {\text{u}}_{\text{n}} - {\text{v}}_{\text{n}} } \right)$$
3. 12 $${\text{Compute }} {\text{v}}_{{{\text{n}} + 1}} = {\text{P}}_{{\mu {\text{B}}_{\text{G}} }} \left( {{\text{f}} - {\text{u}}_{\text{n}} - {\text{w}}_{{{\text{n}} + 1}} } \right)$$
4. 13 $${\text{u}}_{{{\text{n}} + 1}} = {\text{f}} - {\text{v}}_{{{\text{n}} + 1}} - {\text{w}}_{{{\text{n}} + 1 - }} P_{{\lambda {\text{B}}_{\text{G}} }} \left( {{\text{f}} - {\text{v}}_{{{\text{n}} + 1}} - {\text{w}}_{{{\text{n}} + 1}} } \right)$$
5. 14 $${\text{If}}(\hbox{max} \left( {\left| {{\text{u}}_{{{\text{n}} + 1}} - {\text{u}}_{\text{n}} } \right|,\left| {{\text{v}}_{{{\text{n}} + 1}} - {\text{v}}_{\text{n}} } \right|,\left| {{\text{w}}_{{{\text{n}} + 1}} - {\text{w}}_{\text{n}} } \right| \le \varepsilon )} \right)$$

Or if we performed Nstep iterations, then stop the algorithm, else jump to step 2.

In [23], the authors replace ${\text{P}}_{{\delta {\text{B}}_{\text{E}} }} \left( {{\text{f}} - {\text{u}} - {\text{v}}} \right)$ by f − u − v − W_ST_ (f − u − v, δ) where W_ST_ (f − u − v, δ) stands for the wavelet soft thresholding of f – u − v with threshold δ defined by:

15 $${\text{S}}_{\delta } \left( {{\text{d}}_{\text{i}}^{\text{j}} } \right) = \left\{ {\begin{array}{ll} {{\text{d}}_{\text{i}}^{\text{j}} - \delta_{\text{sign}} \left( {{\text{d}}_{\text{i}}^{\text{j}} } \right)} & {{\text{if\,}}\left| {{\text{d}}_{\text{i}}^{\text{j}} } \right| > \delta } \\ 0 & {{\text{if\,}} \left| {{\text{d}}_{\text{i}}^{\text{j}} } \right| \le \delta } \\ \end{array} } \right\}$$

where d_i_^j^ is the wavelet coefficient, j the resolution and i ∊ {x,y, xy}

**Fig. 1:**
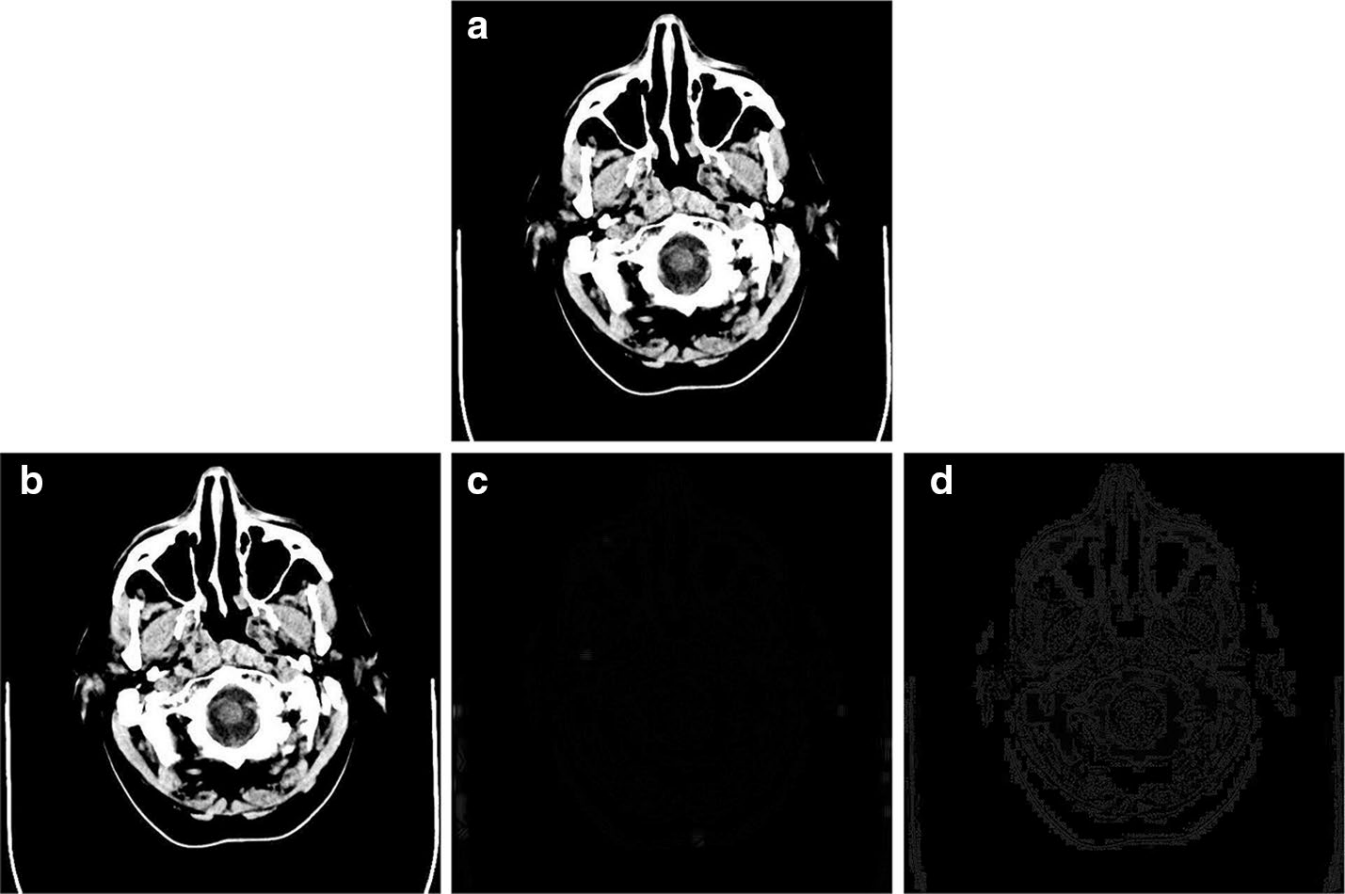
The original image (**a**) is decomposed using PDE decomposition into its structure (**b**), texture (**c**), and noise component (**d**)

Figure 1 shows the application of grayscale decomposition model of Aujol and Chambolle into an image.

### The discrete wavelet transform (DWT)

The discrete wavelet Transform (DWT) is used to decompose an image into different frequency called subbands. At the first level, the image is decomposed into four sub-bands: low frequency band (LL), high frequency band (HH), low–high frequency band (LH), and high-low frequency band (LH). The LL sub-band can further be decomposed to obtain another level of decomposition [24, 25].

DWT is much preferred in digital image watermarking due to its spatial/frequency characteristics which resemble to human visual system, so that watermark imperceptibility and robustness can be improved [26, 27]. Al Haj et al. demonstrated that in order to obtain the best compromise between imperceptibility and robustness, the watermark is to be embedded in the middle frequency sub-bands LHx and HLx [24–26].

### The discrete Fourier transform (DFT)

Given an image f(x,y)of size MxN, the DFT is defined by:

16 $$F\left( {u,v} \right) = \frac{1}{MN}\mathop \sum \limits_{x = 0}^{M - 1} \mathop \sum \limits_{y = 0}^{N - 1} f\left( {x,y} \right)e^{{ - j2\pi (\frac{ux}{M} + \frac{vy}{N})}}$$

The formula can also be written as follows:

17 $$F\left( {u,v} \right) = R\left( {u,v} \right) + jI(u,v)$$

where R(u,v) and I(u,v) denotes the real and imaginary parts of the Fourier transform.The polar representation of the Fourier transform is given by:

18 $$F\left( {u,v} \right) = \left| {F(u,v)} \right|e^{j\emptyset (u,v)}$$

where |*F*(*u*, *v*)| represents the magnitudes and ∅(*u*, *v*) denotes the phase, which are respectively given by:

19 $$\left| {F(u,v)} \right| = \left[ {R^{2} \left( {u,v} \right) + I^{2} \left( {u,v} \right)} \right]^{1/2}$$

20 $$\emptyset \left( {u,v} \right) = tan^{ - 1} \left[ {\frac{I(u,v)}{R(u,v)}} \right]$$

The inverse DFT (IDFT) is given by:

21 $$f\left( {x,y} \right) = \frac{1}{MN}\mathop \sum \limits_{x = 0}^{M - 1} \mathop \sum \limits_{y = 0}^{N - 1} F\left( {u,v} \right)e^{{ - j2\pi \left(\frac{ux}{M} + \frac{vy}{N}\right)}}$$

Selecting the DFT domain to embed the watermark has a certain number of advantages for rotation, scaling and translation (RST) invariance as well as watermark robustness against common signal processing. DFT offers the possibility of embedding watermark either in the magnitude or the phase of the DFT coefficients [20]. Phase-based watermarking shows better robustness against attacks. This is because the phase contains important information of the image [28]. Whereas embedding the watermark in the magnitude part of DFT generates lower visual distortion, as this component contains insignificant information of the image [20, 21, 28].

## The proposed algorithm

Developing an efficient and suitable watermark embedding scheme for medical images and which address the following criteria simultaneously: (1) minimum distortion, (2) robustness against attacks, (3) authenticity, (4) and integrity is a challenging task.

1. The lower level of embedding distortion is required to ensure that the watermarked image can be accepted by the medical professionals for any medical or clinical uses. Assuming that, a cover image is made up of many sub-images (regions). Thus, different regions usually have different capacities for hiding the message. Therefore, deciding how to select the regions for embedding watermark is the key issue in watermarking. Regarding the PDE approach, we tend to select the texture and the noise components as the region for embedding the watermarks. This is motivated by the fact that our human vision is sensitive to slight changes in the smooth regions, while it can tolerate more severe changes in the textures, edges and ridges regions. Therefore, it is expected that fewer detectable artifacts would be left in these regions after watermark embedding [29–34].
2. The proposed scheme is designed to be robust against various kinds of attacks using a blind scheme in the DFT/DWT transform domain.
3. The authenticity is achieved by embedding a robust watermark representing the logo of our University.
4. The integrity of the medical images can be achieved by using a local fragile watermark representing the texture component (v).

### Embedding procedure

To insert the watermarks in the host image, the first step is to decompose the image into its structural (u), textural (v), and noise (w) part, then embedding watermarks information inside the texture and noise components. Watermarked image is obtained by adding the structural part to the other watermarked parts.

**Fig. 2:**
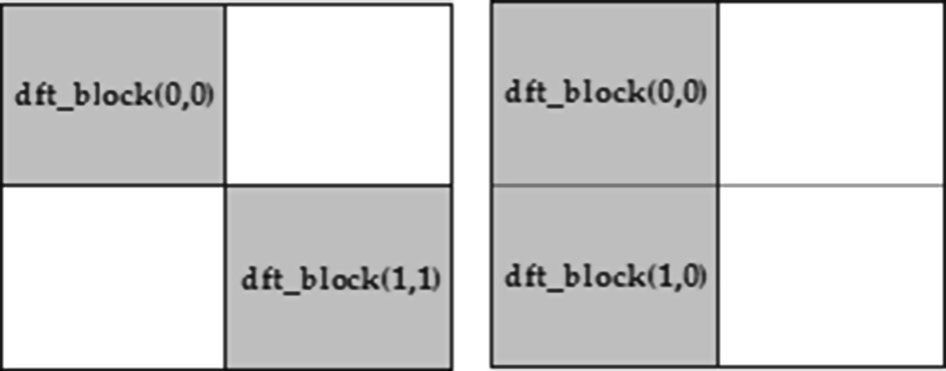
Definition of the chosen DFT coefficients of block 2 × 2

The process of embedding watermarks begins by dividing the host image into 2 × 2 pixels blocks, and then performs a DFT transformation on each block. After that, we select two magnitude coefficients of each 2 × 2 DFT block to embed watermark. With reference to Fig. 2, the chosen coefficients of image block are the DFT coefficients at positions (0, 0), and (1, 0) for the noise component, and the DFT coefficients at positions (0, 0), and (1, 1) for the texture component. To construct the watermarked block, an inverse DFT transformation is performed. This watermarked block then replaces the original block in the host image to form the watermarked image.

**Fig. 3:**
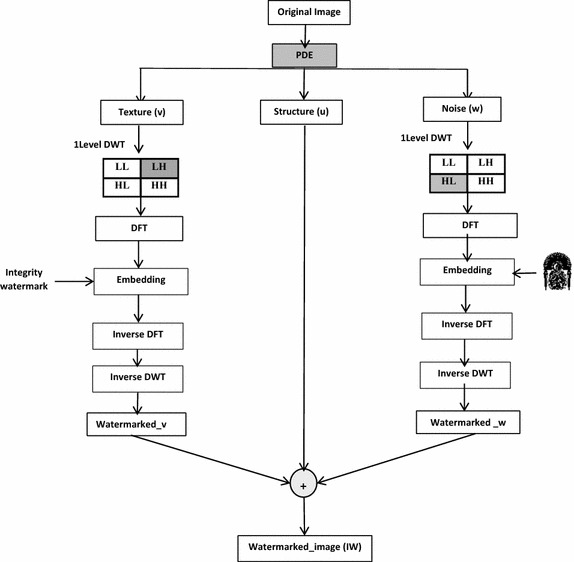
Embedding scheme of the proposed method

The watermark embedding process is represented in Fig. 3, and can be divided in 8 steps:

Step 1 Compute the 1-level DWT for the host image. This operation generates four non-overlapping sub-bands [LL, HL, LH, HH].
Step 2 Segment the selected sub-band X into blocks of 2 × 2.
  where X refers to HL or LH sub-band.
Step 3 Apply forward DFT to each of these blocks. Two DFT coefficients are selected from each block for the embedding process.
Step 4 Reshape the watermark image into a vector of zeros and ones.
Step 5 Embed the binary bits of watermark Wi into the selected DFT coefficients of each block by substituting the watermark bit W(i,j) with the bit of the dft_block (ii, jj): dft_block (ii, jj) = W(i,j).
Step 6 Perform inverse DFT (IDFT) on each block to produce the watermarked block.
Step 7 Reconstruct watermarked blocks to get the final watermarked subband (X′).
  Where X′ refers to the watermarked HL or LH sub-band.
Step 8 Apply the inverse DWT to X′ after the assigned watermark bits have been embedded to form the watermarked image.

### Detection procedure

**Fig. 4:**
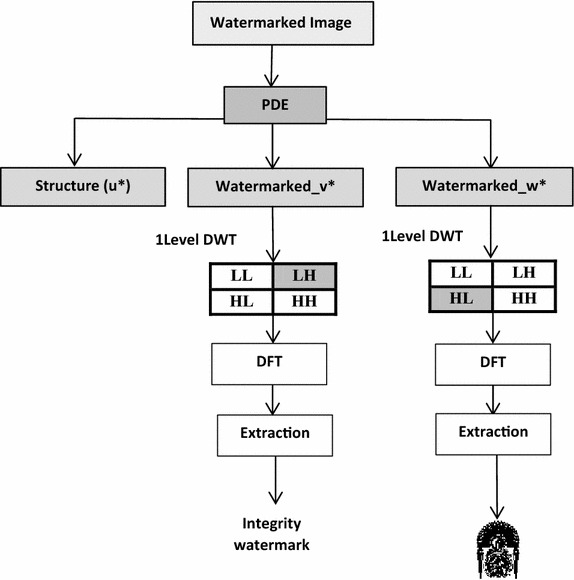
Detection scheme of the proposed method

The watermark detection process is represented in Fig. 4. The first step is to divide the watermarked image into three components: Structure(u*), watermarked_v* and watermarked_w*, and then extracting the watermarks from the watermarked components.

The watermark detection process can be described in the following steps:

Step 1 Compute the 1-level DWT for watermarked image. This operation generates four non-overlapping sub-bands [LL, HL, LH, HH].
Step 2 Segment the selected sub-band X into blocks of 2 × 2. Where X refers to HL or LH sub-band.
Step 3 Apply forward DFT to each of these blocks.
Step 4 Extract the embedded bits of watermark W*(i,j) from the selected frequency coefficients of each block as follows:
  W* (i,j) = dft_block (ii, jj)
Step 5 Form the watermark by concatenation of all bits extracted from each block.
Step 6 Authentication verification. Authentication of the received image can be verified by comparing the original and extracted authentication watermarks.
Step 7 Integrity verification. The integrity of the image is verified by checking if the watermarked image has been modified. It can be verified by comparing the original and extracted local watermarks. If the watermarked image is not modified, both values must be identical. Otherwise, the image is marked as tampered.

## Evaluation and discussion

To check the effectiveness of our proposed method, we have applied the embedding algorithm to a database of 100 grey scale medical images of four modalities: MRI, Ultrasound, X-ray and CT. All images are obtained from [35].

The performance of the watermarking method is investigated by measuring their imperceptible and robust capabilities.

For the imperceptible index, Peek Signal-to-Noise Ratio (PSNR), is employed to evaluate the difference between an original image I and a watermarked image Iw.

The PSNR is defined by the following equation:

22 $${\text{PSNR}} = 10\log_{10} \frac{{255^{2} }}{{ {\text{MSE}}^{{}} }}$$

23 $${\text{MSE}} = \mathop \sum \limits_{{{\text{i}} = 0}}^{{{\text{M}} - 1}} \mathop \sum \limits_{{{\text{j}} = 0}}^{{{\text{N}} - 1}} \left( {\frac{{({\text{I}}_{\text{w}} \left( {{\text{i}},{\text{j}}} \right) - {\text{I}}\left( {{\text{i}},{\text{j}}} \right))^{2} }}{\rm{(M}\times\rm{N)}}} \right)$$

For the robust capability, a measure of Normalized Correlation (NC) between the original watermark W and the corresponding extracted watermark W′ is done. The Normalized Correlation (NC) is defined by the following equation:

24 $${\rm NC} = \frac{{\mathop \sum \nolimits_{i = 1}^{M} \mathop \sum \nolimits_{j = 1}^{N} W\left( {i,j} \right)xW'(i,j)}}{{\left( {\sqrt {\mathop \sum \nolimits_{i = 1}^{M} \mathop \sum \nolimits_{j = 1}^{N} W(i,j)^{2} } } \right)\left( {\sqrt {\mathop \sum \nolimits_{i = }^{M} \mathop \sum \nolimits_{j = 1}^{N} W'(i,j)^{2} } } \right)}}$$

NC value is generally 0 to 1. Ideally NC should be 1.

### Image Watermarking scheme based on PDE, DFT and DWT

#### Imperceptibility

The PSNR is popularly used to measure the similarity between the original image and the watermarked image. While higher PSNR usually implies higher fidelity of the watermarked image. Generally, if PSNR value is greater than 35 dB, the watermarked image is within acceptable degradation levels, and the watermark is almost invisible to human visual system.

**Fig. 5:**
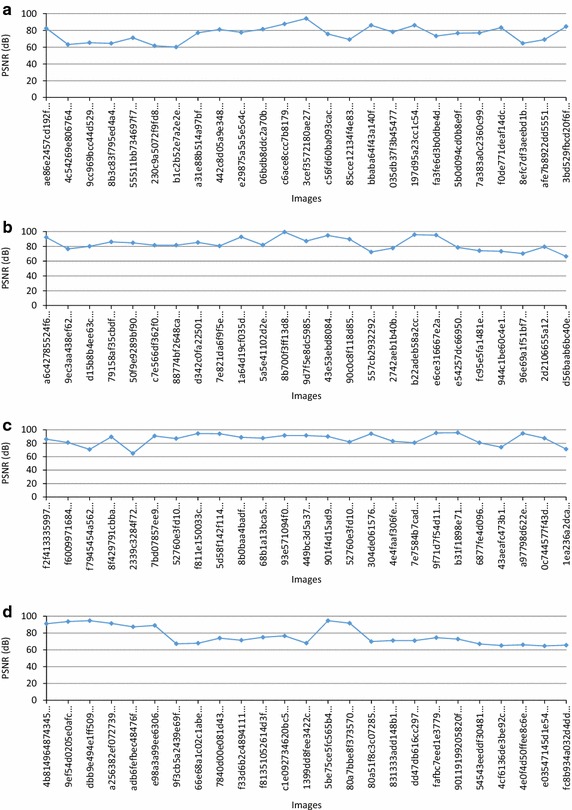
Curve of Peak Signal to Noise Ratio (PSNR) in dB for different **a** CT images, **b** X-Ray, **c** MRI and **d** Ultrasound images

To check the imperceptibility of our method, the PSNR value between the original image I and the corresponding watermarked Iw for different modalities of images is computed. The results obtained are reported in Fig. 5a–d.

The highest PSNR value reached over the 100 images is 94,746 dB, while the lowest PSNR value is 60,1272 dB, which demonstrates the higher imperceptibility nature of the proposed method.

#### Robustness of the watermark

**Fig. 6:**
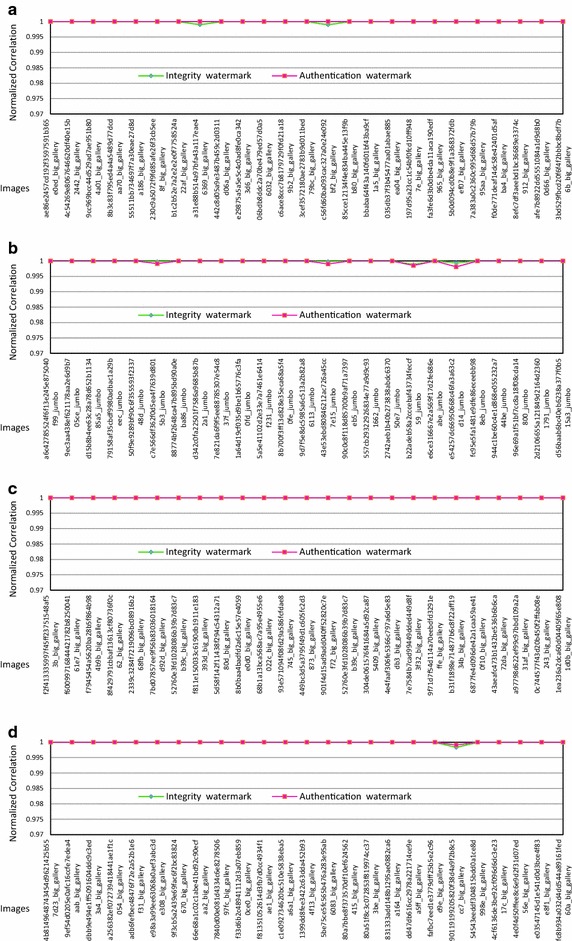
Curve of Normalized correlation for different **a** CT, **b** X-Ray, **c** MRI images and **d** Ultrasound for different watermarks

To check the robustness of the extracted watermarks in attack free case, the Normalized Correlation (NC) between the original watermark and the corresponding extracted watermark for 100 images is computed. The results obtained are reported in Fig. 6a–d.

We get a NC value greater than or equal to 0.998. This indicates that the extracted watermark is very similar to the original watermark for all cases.

### Comparative analysis

#### Robustness in attack free case

**Fig. 7:**
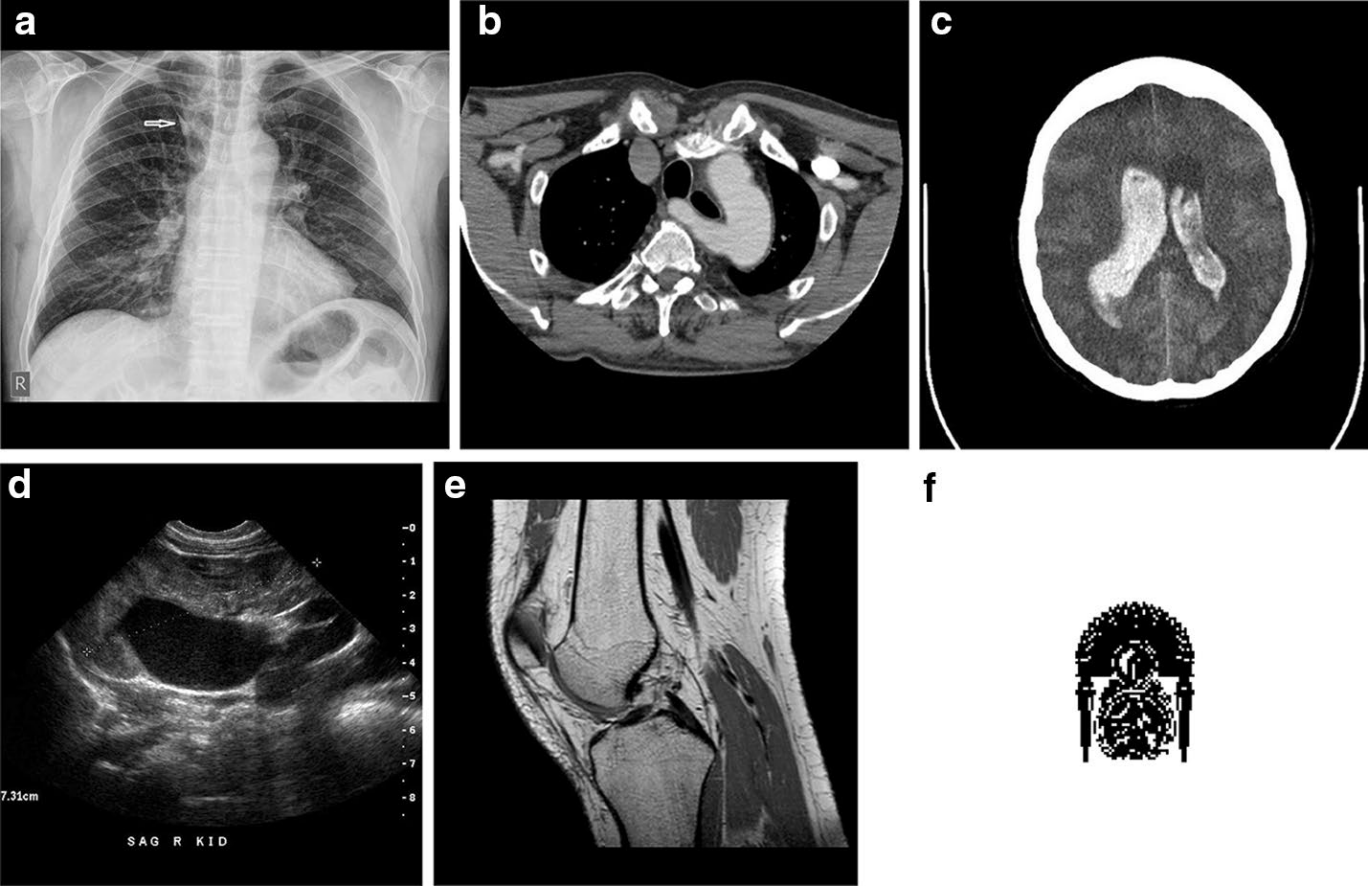
**a**–**e** display the five examined images, **f** Authentication watermark representing the Logo of our University

**Fig. 8:**
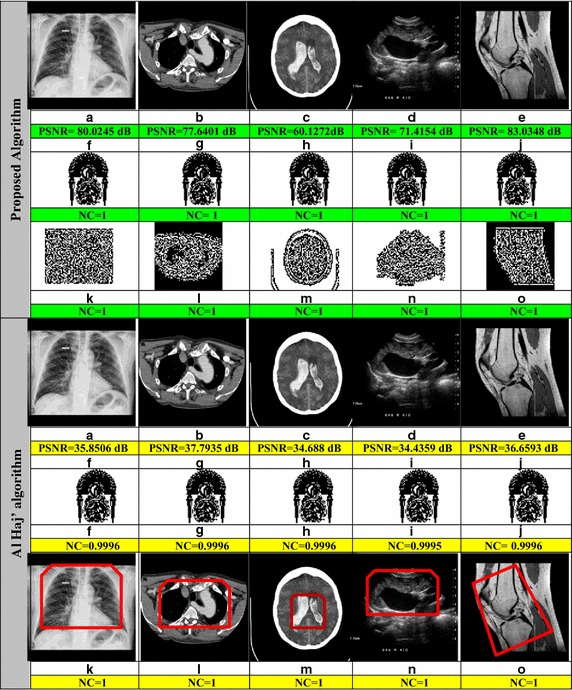
**a**–**e** Watermarked images after embedding the watermarks. **f**–**o** Extracted watermarks from **a**–**e**, respectively

At the first stage, we compare the performance of our proposed method, with the earlier work from Al Haj [12] without the presence of any attack. Experiments have been conducted on five images. Figure 7a–e display the five examined images. Additionally, 64 × 64 binary image, as shown in Fig. 7f is taken as the authentication watermark of images. The results obtained have been shown in Fig. 8. They are presented in terms of Peak Signal to Noise Ratio (PSNR) and Normalized Correlation (NC).

As can be seen from Fig. 8, the proposed scheme provides highest PSNR value compared to Al Haj model [12]. Regarding the NC values, they are almost the same.

Thus the results clearly indicate the imperceptibility and the robustness of the present method in attack free case.

#### Robustness against attacks

In this section, we evaluate the proposed method against attacks. Several image manipulation techniques were used to distort the watermarked images.

Tables 1, 2 show the variation of Normalized Correlation (NC) between the original watermark and the corresponding extracted watermark for some images, after the watermarked images were subjected to Salt and Pepper noise and Gaussian noise attacks respectively.

**Table 1:**
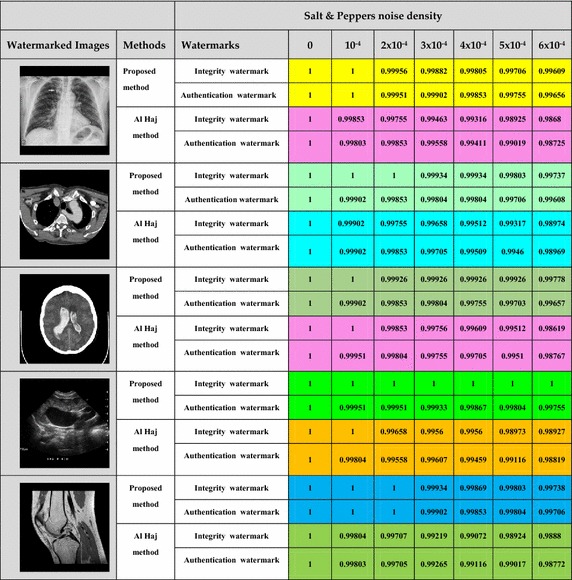
Variation of Normalized Correlation against varying density of Salt and Pepper noise for different cover images

**Table 2:**
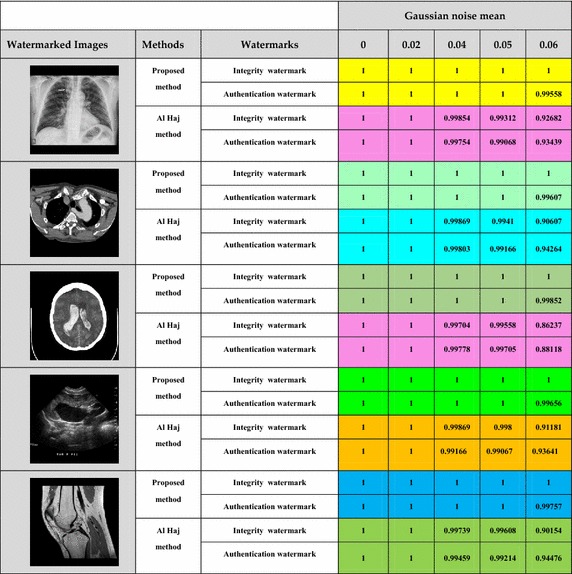
Variation of Normalized Correlation against various density of gaussian noise for different Cover Images

#### Robustness to salt and pepper attack

The robustness of the proposed technique against Salt and Pepper attack is evaluated. The results obtained are reported in Table 1.

As shown in Table 1, in which we analyzed the variation of the Normalized Correlation against varying density of Salt and Pepper. For the proposed technique, we observe that the Normalized Correlation is still high and almost unchanged against an increase of the density of noise.

However, in the case of the Al Haj’s method, we observe that the Normalized Correlation value decreases significantly as the density of noise increases, and that causes a deterioration of detection performance.

##### Robustness to Gaussian noise attack

The performance of the proposed technique against Gaussian noise attack is also evaluated. The experimental results have been shown below in Table 2.

The proposed scheme shows a higher and almost unchangeable Normalized Correlation value for all cases, indicating the highly robust nature of our algorithm against Gaussian noise, and it can be inferred that, Al Haj’s scheme cannot fully resist to higher density of noise.

#### Tamper detection and recovery

In order to demonstrate the tamper localization and recovery function, tampered images were created by modifying some pixel values in the watermarked image. The results obtained are reported in Table 3.

**Table 3:**
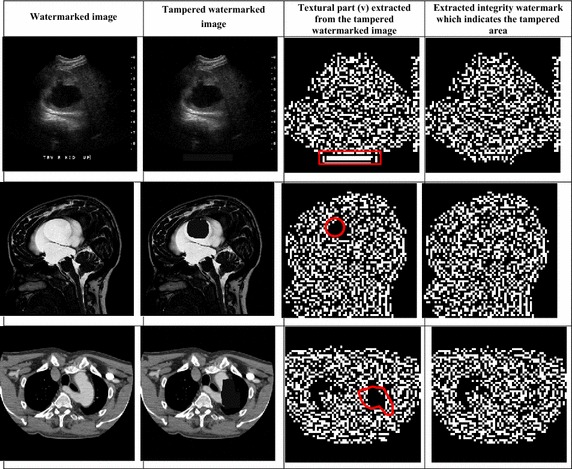
Tamper detection and recovery

Results show that the scheme is capable of detecting and localizing the various types of tampering. Tampered areas are recovered using the compressed texture component (v).

## Conclusion and discussions

Recently, Al-Haj et al. have proposed an interesting ROI/RONI region based watermarking scheme in the frequency and spacial domains. In which, The ROI is watermarked in the spatial domain; whereas, the RONI is watermarked in the frequency domain using a DWT-SVD hybrid transform. The algorithm seems to meet the security requirements of any effective medical image watermarking scheme. For authenticity, the algorithm uses a robust watermark representing the hospital logo watermark. For integrity verification, the algorithm uses a sequence of randomly generated local fragile watermarks to identify and localize tampered blocks. However, the quality of the watermarked images as well as the ROI/RONI selection needs to be improved.

In this work, we aimed to further improve the method of Al-Haj and co-workers. We propose a new watermarking algorithm for multimodal medical images based on PDE, DWT and DFT.

To determine the effectiveness of our proposed method, a number of experiments have been conducted. Based on our experimental results and the comparison between the proposed method and Al Haj’s method we could show that our method based on PDE, DWT and DFT provides highest performance compared to Al Haj’s method.

## Footnotes

1 BV is the space of bounded variations functions. This space is widely used in image processing because it is a good candidate to modelize structures in images.

2 The space of oscillating functions, which contains signals with large oscillations, and thus in particular textures

3 E is a Banach space to model very oscillating patterns.

4 The Legendre-Fenchel transform of F is given by $F^{*} (v) = sup_{u} \left( \langle{u,v\rangle_{{L^{2 - F\left( u \right) } }} } \right)$ where $\langle{.,.}\rangle$_L_^2^ stands for L² inner product.

5 $$\lambda {\rm B}_{\rm G} =\{ {\rm f} \in {\rm G} / {\|f \|}_{\rm G} \leq \lambda \}.$$

6 $$\mu {\text{B}}_{\text{G}} = \left\{ {{\text{v}}\varepsilon {\text{G}}/{\text{v}}_{\text{G}} \le \mu } \right\}.$$

7 $$\delta {\text{B}}_{\text{E}} = \left\{ {{\text{w}}\varepsilon {\text{G}}/{\text{w}}_{\text{E}} \le \delta } \right\}.$$
